# A comparative global phosphoproteomics analysis of obinutuzumab (GA101) versus rituximab (RTX) against RTX sensitive and resistant Burkitt lymphoma (BL) demonstrates differential phosphorylation of signaling pathway proteins after treatment

**DOI:** 10.18632/oncotarget.23040

**Published:** 2017-12-09

**Authors:** Aradhana Awasthi, Delphine C.M. Rolland, Janet Ayello, Carmella van de Ven, Venkatesha Basrur, Kevin Conlon, Damian Fermin, Matthew J Barth, Christian Klein, Kojo S.J. Elenitoba-Johnson, Megan S. Lim, Mitchell S. Cairo

**Affiliations:** ^1^ Department of Pediatrics, New York Medical College, Valhalla, NY, USA; ^2^ Department of Medicine, New York Medical College, Valhalla, NY, USA; ^3^ Department of Pathology, New York Medical College, Valhalla, NY, USA; ^4^ Department of Microbiology & Immunology, New York Medical College, Valhalla, NY, USA; ^5^ Department of Cell Biology & Anatomy, New York Medical College, Valhalla, NY, USA; ^6^ Department of Medicine, Roswell Park Cancer Institute, Buffalo, NY, USA; ^7^ Roche Pharmaceutical Research & Early Development, Roche Innovation Center Zurich, Schlieren, Switzerland; ^8^ Department of Pathology, University of Michigan Medical School, Ann Arbor, MI, USA; ^9^ Department of Pathology and Laboratory Medicine, University of Pennsylvania Perelman School of Medicine, Philadelphia, PA, USA

**Keywords:** obinutuzumab, rituximab, Burkitt lymphoma, proteomics, resistant

## Abstract

We recently demonstrated that obinutuzumab (GA101), a novel glycoengineered type II CD20 Ab compared to rituximab (RTX) mediates significantly enhanced antibody-dependent cell cytotoxicity (ADCC) *in vitro* and increased overall survival in a Burkitt lymphoma (BL) xenograft non-obese diabetic severe combined immunodeficiency gamma (NSG) model. In this study we compared the phosphoproteomic changes by pathway analysis following obinutuzumab vs RTX against RTX-sensitive (Raji) and -resistant BL (Raji4RH). Phosphoproteomic analyses were performed by mass-spectrometry (MS)-based label-free quantitative phosphoproteomic profiling. We demonstrated that 418 proteins in Raji and 377 proteins in Raji 4RH, were differentially phosphorylated (>1.5-fold) after obinutuzumab vs. RTX. Proteins that were significantly differentially phosphorylated included the B cell antigen receptor (BCR) (PLCG2, BTK and GSK3B), Fc gamma phagocytosis (FCRG2B, MAPK1, PLCG2 and RAF1), and natural killer cell-mediated cytotoxicity (MAPK1, RAF1, PLCG2 and MAPK3) signaling pathways. Differential phosphorylation of BCR or cytotoxicity pathway proteins revealed significant up-regulation of BTK, PLCY2 and ERK1/RAF1 after obinutuzumab compared to RTX. Silencing of PLCG2 in the BCR and MAPK1 in the cytotoxicity pathway significantly increased BL proliferation and decreased BL cytotoxicity after obinutuzumab compared to RTX. These results in combination with our previous results demonstrating a significant improvement in *in vitro* BL cytotoxicity and *in vivo* BL survival by obinutuzumab compared to RTX may in part be due to differential effects on selected BL protein signaling pathways.

## INTRODUCTION

Non-Hodgkin lymphoma (NHL) is the seventh most common cause of cancer in the United States [1]. While Burkitt lymphoma (BL) represents less than 1% of all adult NHL, it accounts for approximately 40% of all malignant NHL in children and adolescents [2, 3]. CD20, which is expressed on normal and malignant mature B-cells, has proven to be an excellent target for immunotherapeutic approaches in B-cell hematological malignancies [4]. CD20 has also been demonstrated to be expressed in ≥ 98% of mature B-cell lymphomas in children and adolescents including BL/ leukemia and approximately 35-45% of pediatric precursor B-cell acute lymphoblastic leukemia (pre-B-ALL) [5, 6]. Based on their mechanism of action, anti-CD20 monoclonal antibodies (mAbs) may be divided into two major sub-types, type-I and type-II. Both type-I and type-II anti-CD20 antibodies can also be distinguished by their ability to redistribute CD20 into lipid rafts within the cell membrane [7, 8].

The majority of the anti-CD20 antibodies in clinical use or in investigation are within the type-I category, including rituximab (RTX), veltuzumab, ocrelizumab, ublituximab and ofatumumab [9-11]. The mouse/human chimeric type-I, CD20 mAb, RTX, was the first approved therapeutic mAb against B-cell hematological malignancies and since then has become one of the most important treatment modalities for B cell malignancies including most subtypes of adult indolent B-cell lymphomas [12]. The incorporation of RTX in the management of B-cell NHL (B-NHL) in children and adolescents has improved the response rate, progression free survival (PFS, 90%), and overall survival (OS) [13]. Further, RTX alone and in combination with chemotherapy has been used successfully in adults with indolent B-NHL and diffuse large B-cell lymphoma (DLBCL) [12, 14-19], and also, demonstrated to be safe in children and adolescents with mature B-NHL including BL [20-22].

Despite the successful clinical use of RTX, the underlying mechanisms associated with the efficacy of RTX remain elusive. Further, it is not clear why subsets of patients are initially unresponsive and many responding patients become refractory and resistant to further RTX administration [23, 24]. One of the mechanisms of failure following RTX treatment is the development of resistance to RTX [24, 25]. Antigen escape is not a major mechanism of RTX resistance. However, mechanisms of tumor resistance to anti-CD20 mAb therapy have been postulated to be either tumor-related, (e.g. down-regulation of targeted extracellular CD20 antigen, acquisition of a protective phenotype (by up-regulation of complement inhibitory proteins, etc.) or host-related factors (e.g. Fcγ receptor polymorphisms) [26]. Attempts have been made to develop model systems to investigate the mechanism of resistance to RTX with the hope to apply these findings and to generate new therapeutic approaches to circumvent RTX resistance [24].

Obinutuzumab is a humanized type-II CD20 targeted mAb recognizing a unique CD20 type-II epitope [27]. One of the possible mechanisms of RTX resistance could be the loss of surface CD20 expression after RTX therapy. Normal and malignant B cells express the inhibitory Fc receptor FCγRIIb that binds to the Fc domain of RTX. Together the CD20/RTX/FcγIIb complex is then internalized in to CD20 positive B-NHL cells and subsequently degraded. Thus, it is possible that FcγIIb expression on malignant B cells might serve as a biomarker for response to RTX. Alternatively, internalization may be overcome by utilizing the obinutuzumab type-II anti-CD20 antibody, which seems to be internalized far less than type-I antibody or possibly by combining RTX with an anti-FcγIIb inhibitor [15, 28].

The fragment crystallizable (Fc) region of this antibody has been glycoengineered leading to bisected, afucosylated Fc region carbohydrates resulting in enhanced affinity for the human FcγRIIIa receptor on human effector cells, such as natural killer (NK) cells, macrophages and dendritic cells. *In vitro* studies using obinutuzumab have demonstrated significantly increased antibody-dependent cell cytotoxicity (ADCC) and superior direct cell death compared to RTX [29]. Obinutuzumab compared to RTX has also been shown to enhance whole blood B-cell depletion in both healthy volunteers and in patients with chronic lymphocytic leukemia (CLL) [30]. Studies in human indolent CD20+ B-NHL xenografts in severe combined immunodeficiency (SCID) beige mice have demonstrated significantly greater efficacy in reducing tumor size, inducing remission and improving survival following obinutuzumab compared to similar doses of RTX [29]. Our group recently demonstrated that equal doses of obinutuzumab compared to RTX significantly enhanced OS and reduced tumor burden in RTX-sensitive and -resistant BL xenografted non-obese diabetic severe combined immunodeficiency gamma (NSG) mice [24, 31]. We also observed significantly enhanced cell death and ADCC *in vitro* against BL RTX-sensitive and -resistant cell lines. Most recently, obinutuzumab in combination with chlorambucil compared to the standard therapy with chlorambucil and RTX has been demonstrated to improve overall response and PFS in patients with CLL [32].

Furthermore, our group has successfully utilized proteomic profiling to develop biomarkers with potential clinical usefulness in several cancer types [33, 34]. Abnormal protein phosphorylation with distinct signatures in B-cell lymphoma has been reported by our group and others [35]. Previous work from our group [33] demonstrated that Epstein Barr virus (EBV)+ Raji cells (BL) overexpressed a number of phosphoproteins compared to normal B-cells including SYNCRIP, HSP90ABI, SLC3A2 (fold change 2.6, 3.9 and 5.0). Tandem mass spectrometry (MS) has become sufficiently sensitive and is a robust tool for proteomic studies, as thousands of proteins can be confidently identified in a single step. Furthermore, tandem mass spectrometry (MS/MS) allows for the definitive identification of protein sequences and site of phosphorylation as described by our group and others [35, 36].

This study focuses on the differential phosphoproteomic profiling of RTX-sensitive and -resistant BL cell lines treated with obinutuzumab vs RTX. To our knowledge this is the first study to compare phosphoproteomic profiling between two CD20 mAbs (RTX compared to obinutuzumab) in RTX-resistant and -sensitive BL cells.

## RESULTS

### Identification and quantification of phosphorylated proteins in RTX-sensitive and -resistant BL cell lines after obinutuzumab compared to RTX treatment

**Figure 1:**
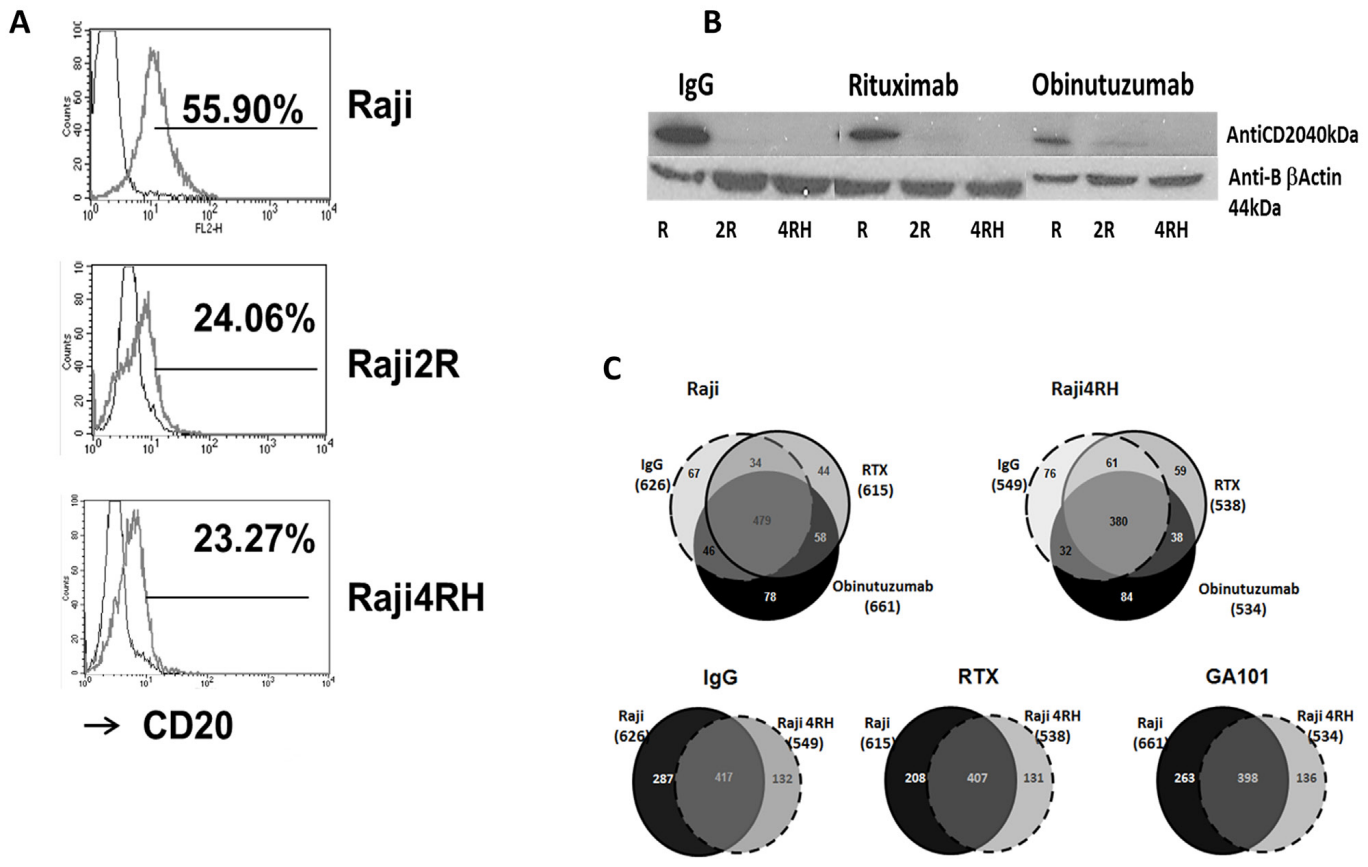
Identification and quantification of phosphorylated proteins in RTX-sensitive and -resistant BL cell lines after obinutuzumab vs. RTX **(A)** CD20 expression: RTX-sensitive (Raji), RTX-resistant (Raji2R and Raji4RH) cell lines were stained with anti-CD20-PE antibody and surface expression of CD20 was determined by flow cytometry (52.3±8.3% vs. 23.9±1.4%, p=0.00003). **(B)** CD20 expression after obinutuzumab vs. RTX treatment: RTX-sensitive (Raji) and RTX-resistant (Raji2R and Raji4RH) cell lines were treated with 100ug/ml of obinutuzumab (obit), RTX and isotype control IgG for 24 hrs. Cells were lysed and total proteins (40 μg) were suspended in Laemmli sample buffer, boiled, and subjected to SDS polyacrylamide gel electrophoresis on 10% gels for CD20 expression (N=3). **(C)** Protein identification by phosphoproteomic analysis: Six milligrams of protein from each condition were digested by trypsin and peptides were subjected to phosphopeptide enrichment using metal oxide affinity chromatography (MOAC) and immunoprecipitation for phosphoproteiomics analysis. An LTQ Orbitrap XL in-line with a Paradigm MS2 HPLC was employed for acquiring high-resolution MS and MS/MS data that were searched with the Swissprot Human taxonomic protein database (N=3).

CD20 expression was first verified in each BL cell line (Raji and Raji4RH) by flow cytometry using CD20-PE antibody as described previously (Figure 1A) [31]. There was significantly less CD20 expression in the RTX-resistant cell line (23.98±1.45%) compared to the RTX-sensitive cell line, (52.36±8.39%, p=0.00003, Figure 1A).

There was a significant decrease in CD20 expression by western blot in both BL rituximab sensitive and resistant cell lines by both rituximab and obinutuzumab compared to IgG isotype control (Figure 1B).

Raji and Raji4RH were treated with obinutuzumab or RTX (100μg/ml for 24h) or isotype control for MS-based label-free quantitative phosphoproteomic profiling. Six milligrams of protein from each condition were digested by trypsin and peptides and subjected to phosphopeptide enrichment using metal oxide affinity chromatography (MOAC) and immunoprecipitation. Two independent phosphopeptide enrichments were performed for each experimental condition and high mass accuracy MS was performed in technical triplicates. There were 978 unique phosphorylated proteins identified. The analysis of the MOAC enrichments led to the identification of 957 unique phosphorylated proteins and the p-Tyr-IP enrichments led to the identification of 42 unique phosphorylated proteins. Of note, 20 phosphorylated proteins were identified in both MOAC and p-Tyr-IP enrichments. In detail, 626 phosphorylated proteins were identified in the Raji cell line when treated with the anti-CD20 isotype control (immunoglobulin G [IgG]) while 615 and 661 phosphorylated proteins were identified after RTX and obinutuzumab treatment, respectively (Figure 1C). For the RTX-resistant cell line Raji4RH, 549 phosphoproteins were identified after IgG treatment while 538 and 534 phosphoproteins were identified after RTX and obinutuzumab treatment, respectively (Figure 1C). As shown in Figure 1C lower half, after the anti-CD20 isotype control (IgG) total 417 phosphoproteins were identified in both Raji and Raji4RH, with 287 phosphoprotein identified in Raji and 131 in identified Raji4RH. The same observation was made after both anti-CD20 treatments. Indeed, after RTX treatment 407 phosphoproteins were identified in both Raji and Raji4RH, with 208 phosphoproteins were identified in Raji and 131 identified Raji4RH. After obinutuzumab treatment, 398 phosphoproteins were identified in both Raji and Raji4RH, with 263 phosphoproteins were identified in Raji, and 136 identified inRaji4RH.

### Distinctive phosphoproteomic signatures discriminate obinutuzumab compared to RTX-mediated signaling in RTX-sensitive (Raji) and RTX-resistant (Raji4RH) BL cell lines

**Figure 2:**
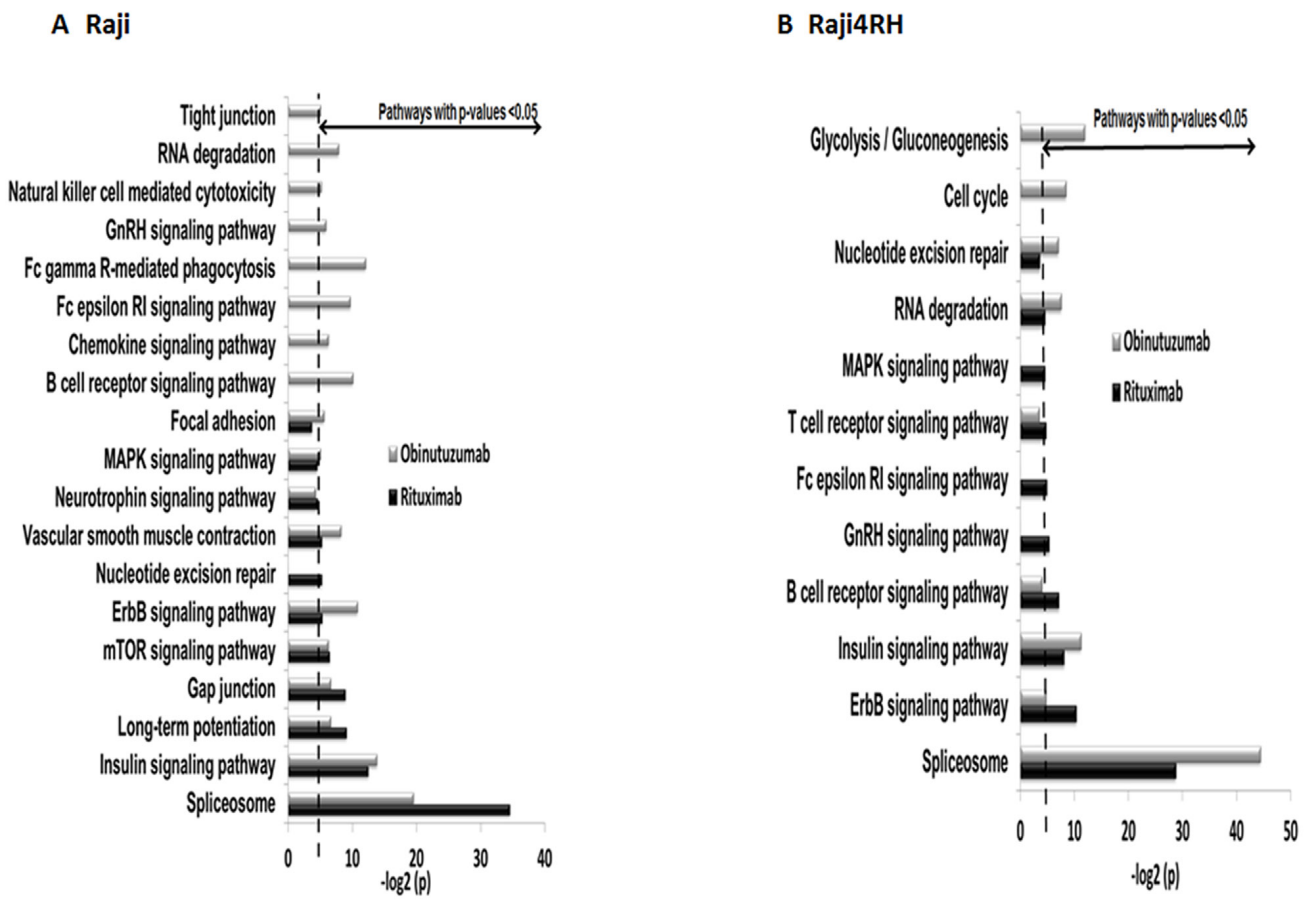
Differential phosphorylation of pathway proteins after RTX vs. obinutuzumab treatment Swissprot human taxonomic protein database identify proteins phosphorylated at serine, threonine and tyrosine residues. Proteins with >1.5 fold increase in phosphorylation between anti-CD20 mAbs (RTX and Obinutuzumab) and IgG treatment in **(A)** Raji and **(B)** Raji4RH (P=0.05) were identified in multiple cellular pathways.

**Figure 3:**
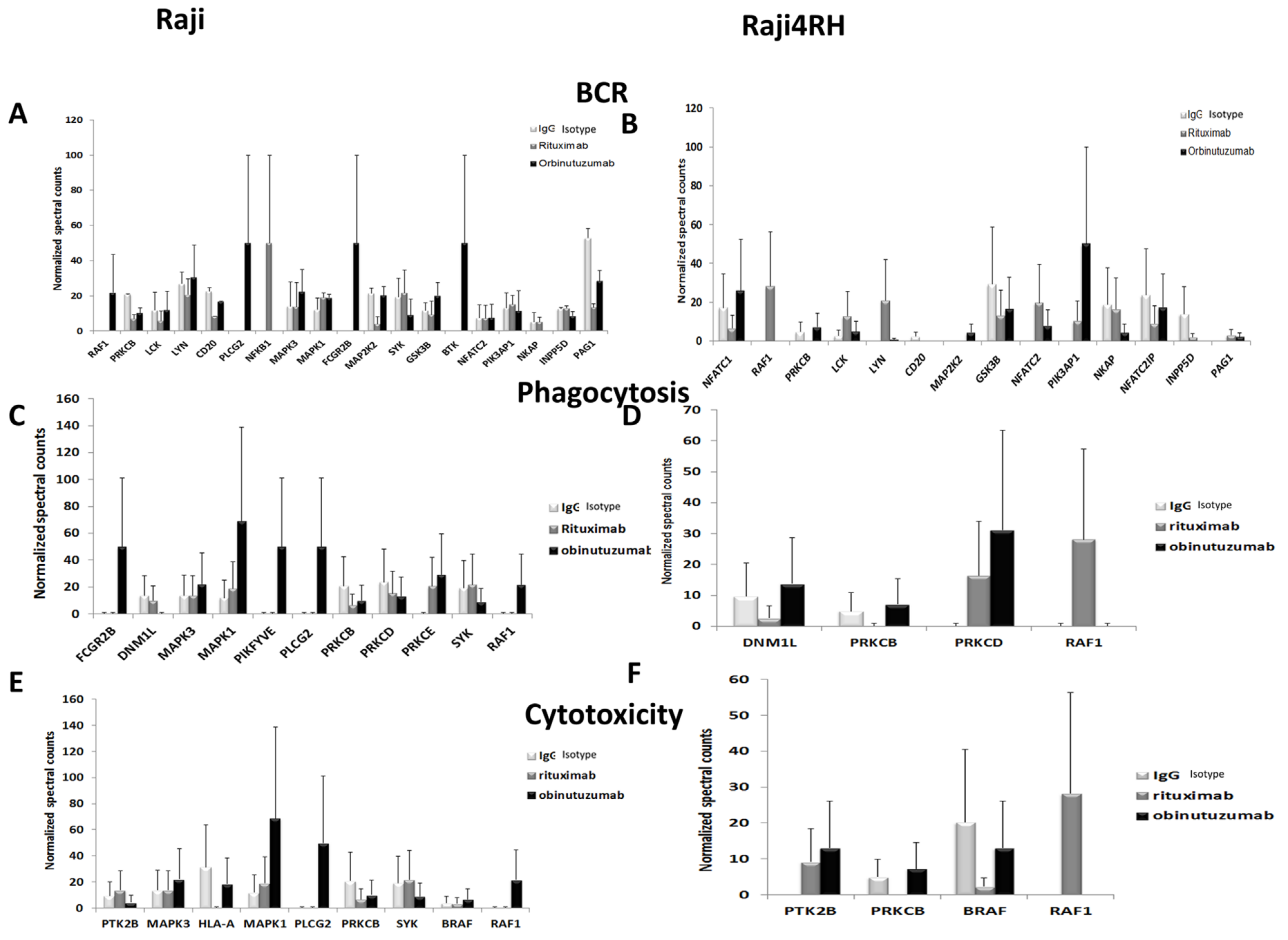
Phosphoproteomic analysis identifies proteins in BCR, phagocytosis and cytotoxicity pathways that are differentially altered after obinutuzumab vs. RTX Mass spectrometry-based phosphoproteomic analysis identifies differences in phosphorylation of proteins that function in the BCR pathway **(A)** Raji **(B)** Raji4RH, phagocytosis **(C)** Raji, **(D)** Raji 4RH and cytotoxicity **(E)** Raji, **(F)** Raji4RH (N=3).

To functionally categorize the phosphoprotein changes induced by RTX compared to obinutuzumab in RTX-sensitive and -resistant BL cell lines, we utilized the Database for Annotation, Visualization and Integrated Discovery (DAVID) software version 6.7. Only proteins with at least 1.5-fold change in their phosphorylation level between anti-CD20 treatment and IgG control were included. As a result, 418 proteins were differentially phosphorylated between IgG and RTX treatments in Raji cell line while 377 were differentially phosphorylated between the same conditions in Raji4RH cell line. The obinutuzumab treatment (in comparison to IgG treatment) resulted in the differential phosphorylation of 440 proteins for Raji cell line and 508 proteins in Raji 4RH cell line. As shown in Figure 2, numerous signaling pathways were significantly (p<0.05) modified after the treatment of Raji (Figure 2A) and Raji4RH (Figure 2B) with both anti-CD20 antibodies, several phosphoproteins in the B cell antigen receptor (BCR) signaling pathway (RAF1, PLCG2, Bruton tyrosine kinase [BTK], Lyn, LCK, FGR2B and PAG1 in Raji as compared to NFATC1, PI3KAP1 and NFTC2IP in Raji4RH, Figure 3A and 3B), Fc gammaR-mediated phagocytosis (FGR2B, MAPK1, PIKFYVE, PLCG2 and RAF1 in Raji and PRKCB, PKCD and RAF1 in Raji 4RH, Figure 3C and 3D) and the NK-cell-mediated cytotoxicity (MAPK1, PLCG2, RAF1 and BRAF1 in Raji compared to PTK2B, BRAF and RAF1 in Raji4RH, Figure 3E and 3F) were changed by obinutuzumab as compared to RTX treatment (Figure 3).

### Obinutuzumab compared to RTX differentially regulate proteins associated with BCR signaling pathway in RTX-sensitive and -resistant cell lines

Our proteomic profiling after obinutuzumab vs. RTX revealed differential phosphorylation of several proteins associated with BCR signaling pathway in Raji. Upregulated hyper-phosphoproteins include PLCG2 (50 fold), BTK (50 fold) and Lyn (30.6 fold) after treatment with obinutuzumab in RTX-sensitive Raji cell lines (Figure 3A), though, GSK3B (20.08 fold) and LCK (11.86 fold) did not increase hyper-phosphorylation of proteins after obinutuzumab treatment. RTX treatment also upregulated some phosphoproteins of the BCR signaling pathway in Raji LYN (20.68, Figure 3A), LCK (5.6 fold, Figure 3A) and PAG1 (13.48 fold, Figure 3A) compared to obinutuzumab. However, we did not observe similar effects in the Raji4RH RTX-resistant cell line for PLCG2 or BTK after obinutuzumab vs. RTX treatment; although, RTX upregulated Lyn and LCK phosphoproteins after RTX treatment in Raji4RH cell lines (0.77 vs 21.0 fold and 5.0 vs. 12.7 fold, Figure 3B).

**Figure 4:**
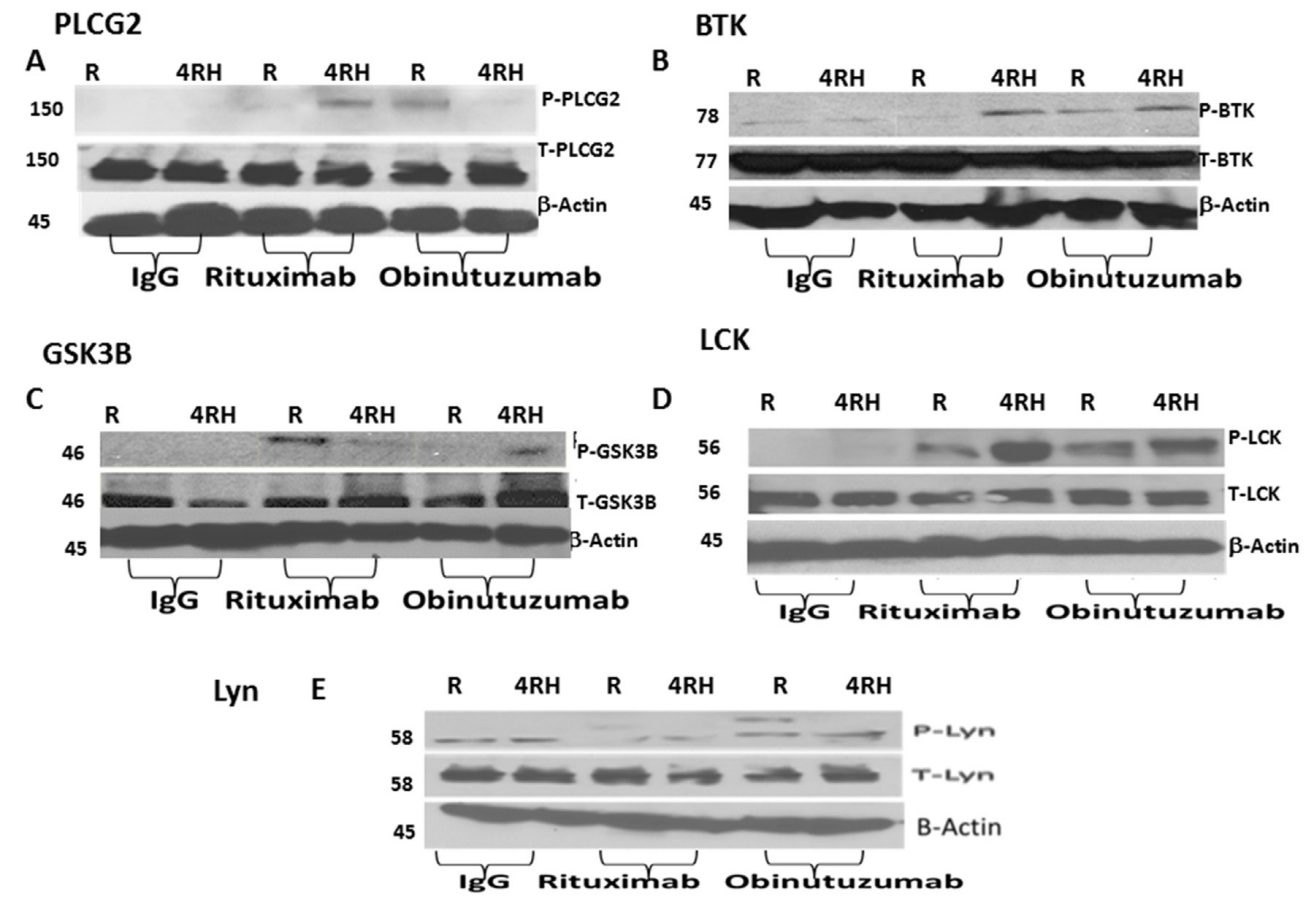
Phosphorylation of proteins involved in BCR signaling pathways are differentially expressed after obinutuzumab (Obit) vs. RTX Differential phosphorylation of proteins was identified by MS-based phosphoproteomic analysis. Phosphorylation of proteins involved in BCR signaling pathways including **(A)** PLCG2, **(B)** BTK, **(C)** GSK3B, **(D)** LCK and **(E)** Lyn in Raji and Raji4RH BL cell lines. Obinutuzumab vs. rituximab treated cells were lysed and subjected to SDS polyacrylamide gel electrophoresis and western blot analyses were performed using antibodies as shown.

We orthogonally validated the MS-based semi-quantitative proteomic results by western blot analysis of selected phosphoproteins. We investigated the expression and phosphorylation status of proteins involved in BCR signaling pathways by western blot (Figure 4A-4E and Supplementary Figure 1). Figure 4 demonstrates the differential phosphorylation of 5 selected proteins (PLCG2, BTK, GSK, LCK and Lyn) in which obinutuzumab upregulates the phosphorylation of PLCG2, BTK and GSK3B in Raji (Figure 4A-4C) but not in Raji4RH (Figure 4A-4C). Phosphorylation of LCK was upregulated by RTX in Raji4RH compared to Raji (Figure 4D). Also in some cases IgG treated cell lines show upregulation of phosphorylation of protein both in Raji vs. Raji4RH (LCK, LYN, Figure 4D-4E) and Raji4RH vs. Raji (GSK3B, Figure 4C).

### PLCG-2 knockdown promotes BL cell proliferation in RTX-sensitive Raji compared to RT-resistant Raji4RH when treated with obinutuzumab compared to RTX

As stated above, obinutuzumab compared to RTX differentially regulates proteins associated with BCR signaling pathway in RTX-sensitive and -resistant cell lines. Here we investigated a major difference in the requirement of the B-cell signaling molecule, PLCG2, for cell proliferation in RTX-resistant as well as -sensitive cell lines. As we observed in Figures 3A, 3B and 4A, P-PLCG2 was hyperphosphorylated in RTX-sensitive Raji cell lines after obinutuzumab treatment. In contrast phosphorylation of PLCG2 was not detectable in RTX-resistant cell lines Raji4RH after obinutuzumab treatment (Figure 3A & 3B, Figure 4A and Supplementary Figure 2A and 2B, p=0.0001). Based on above observations we hypothesized that depletion of PLCG2 in RTX sensitive (Raji) and resistant cells lines (Raji4RH) leads to incapacitating the downstream signaling of obinutuzumab vs. rituximab.

**Figure 5:**
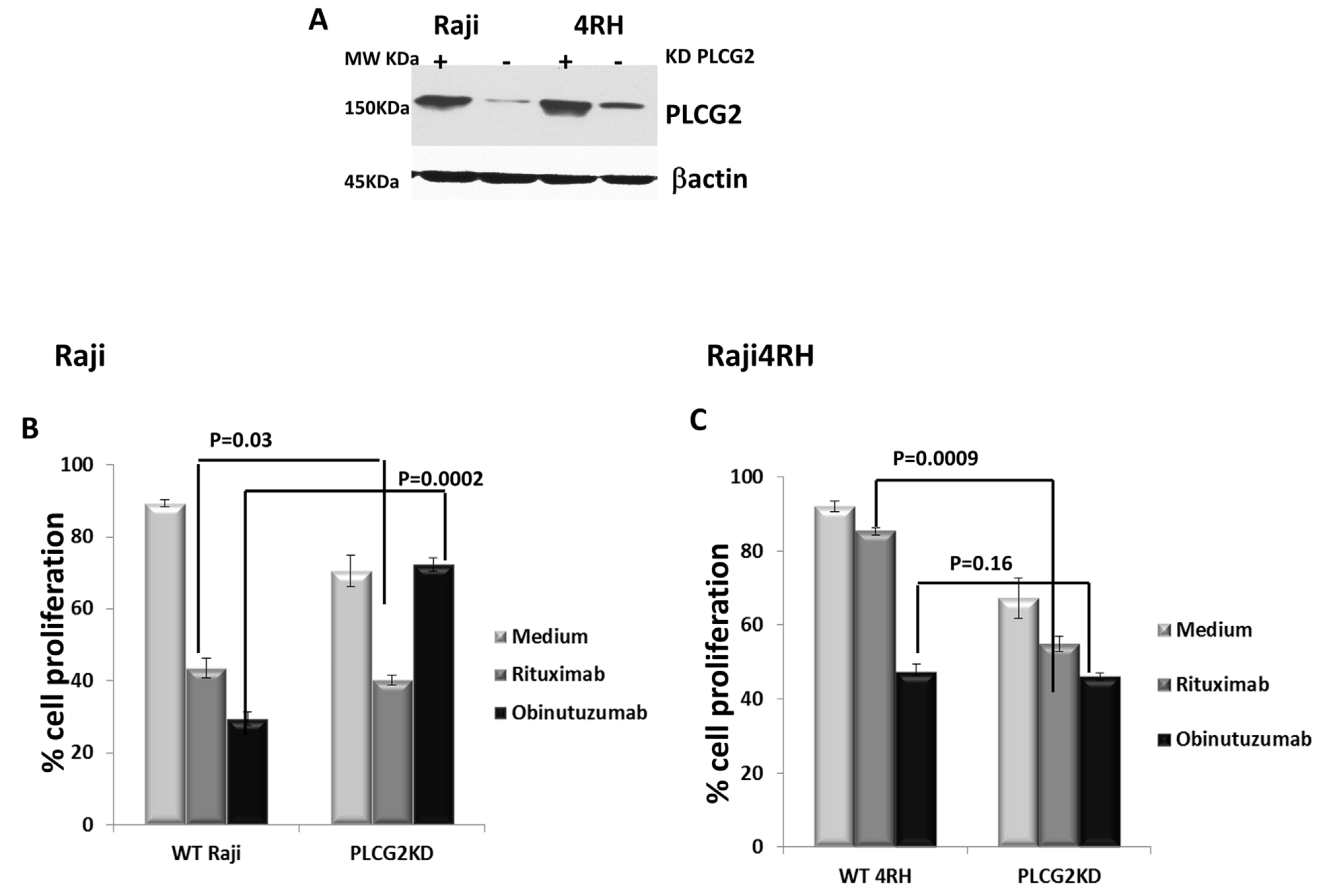

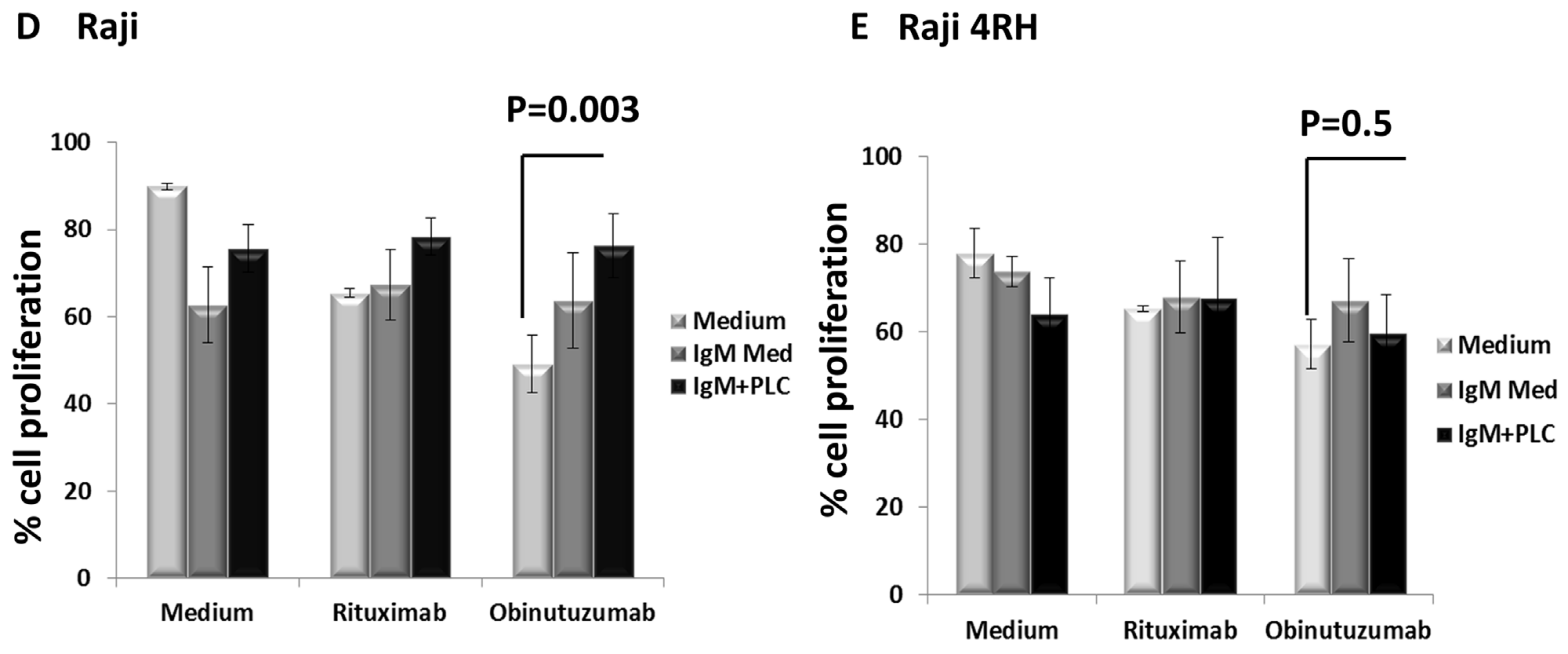
Silencing of PLCG2 significantly increased cell proliferation in Raji (RTX-sensitive) vs. Raji4RH (RTX-resistant) after obinutuzumab (Obit) vs. RTX Silencing of **(A)** PLCG2 and in Raji and Raji4RH cell lines was carried out by SiRNA. Cell proliferation was determine by almar blue dye after PLCG2KD in Raji **(B)**, p=0.0002 vs. 0.03, respectively) and **(C)** Raji 4RH (p=0.16 vs 0.00009, respectively). PLCG2KD cells were treated with (anti-IgM Ab for antigen stimulation with obinutuzumab or rituximab and analyzed by almar blue **(D)** Raji **(E)** Raji4RH. Data were shown in mean±SD. (N=3), (p=0.003 and 0.5, respectively).

To investigate a possible functional role of PLCG2 after obinutuzumab compared to RTX treatment in Raji compared to Raji4RH, we performed PLCG2 depletion by siRNA-mediated knockdown in Raji and Raji4RH. Our *PLCG2* silencing achieved 80-85% knockdown of PLCG2 protein expression by western blot analysis (Figure 5A).

We examined the impact of PLCG2 depletion on BL cell proliferation using the vital dye alamar. *PLCG2* knockdown (PLCG2KD) in Raji resulted in significant increase in cell proliferation after obinutuzumab treatment compared to wildtype Raji (27.6±1.84% vs. 70.4±1.8%, p=0.0002, Figure 5B). PLCG2KD in Raji4RH had no effect on BL cell proliferation after obinutuzumab treatment (p=0.16, Figure 5C). However, depletion of PLCG2 significantly reduced BL cell proliferation of Raji cells after RTX treatment (p=0.03). Similar results have been observed for Raji4RH for which the depletion of PLCG2 significantly increased the cell death when treated with RTX (p=0.0009). These results suggest in part that obinutuzumab reduces cell proliferation through the PLCG2 mediated signaling pathway. Knocking down of PLCG2 significantly enhanced cell proliferation in the RTX-sensitive cell line compared to the RTX-resistant Raji4RH.

Because PLCG2 is involved in the BCR signaling pathway, we evaluated the ability of Raji and Raji4RH cell lines to respond to antigen stimulation when PLCG2 was silenced and treated with obinutuzumab compared to RTX. We demonstrated that PLCG2KD led to a significant increase in cell proliferation in response to antigen stimulation after 48 hrs in Raji treated cells after obinutuzumab compared to RTX. No significant increase in cell proliferation was observed in Raji4RH after antigen stimulation treated with obinutuzumab or RTX (Figure 5D and 5E). BL cell proliferation was significantly increased in Raji (51±5.09% vs. 24±7.1%, p=0.003), after obinutuzumab+PLCG2KD+IgM vs. obinutuzumab+PLCG2KD treatment only. However, we did not observe similar results in Raji4RH (43±8.2% vs. 41±9.2%, p=NS).

To further investigate the extent to which PLCG2 influenced obinutuzumab compared to RTX via a BCR signaling pathway, we analyzed the expression of p-PLCG2 after obinutuzumab treatment in Raji (Supplementary Figure 2A). P-PLCG2 was over-expressed in Raji but not in Raji4RH (Supplementary Figure 2B). Furthermore, the silencing of the PLCG2 in Raji significantly reduced cell death compared to wildtype Raji cells (43.16±0.8 vs. 17.1±5, p=0.0004) (Supplementary Figure 2C). RTX-treated Raji cells had no effect in BL cell death after silencing with PLCG2 (Supplementary Figure 2C). However, we also observed no significant change in cell death after PLCG2 knockdown in Raji4RH (23.5±1.5 vs. 23.3±3.2, p=NS, Supplementary Figure 2D).

### Extracellular signal-regulated kinase (ERK1) silencing by U0126 inhibitor reduces cytotoxicity after obinutuzumab treatment in the Raji BL cell line

**Figure 6:**
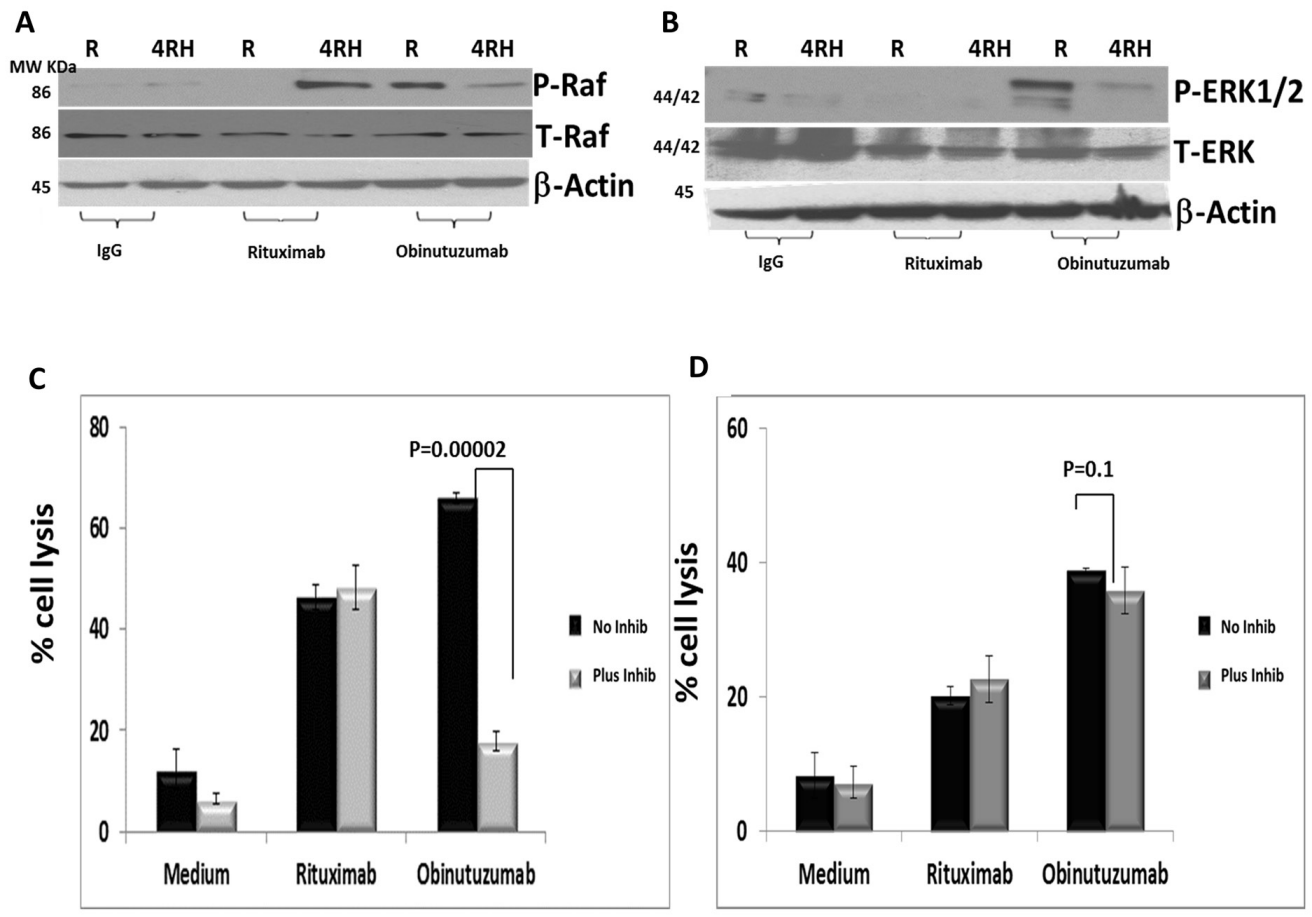
Differential phosphorylation of RAF/ERK1/2 in Raji and RajiRH may contribute to differential effect of ERK1 inhibition on NK-mediated cytotoxicity after obinutuzumab (Obit) vs. RTX Raji vs. Raji4RH were treated with obinutuzumab vs. rituximab and analyzed by western blot for cytotoxicity signaling pathway proteins RAF and ERK1/2 **(A)** RAF total and p-RAF. **(B)** Total ERK total p-ERK1/2. Raji, Raji4RH cells were incubated with MAPK1 inhibitor (10 μM) and subsequently treated with obinutuzumab, rituximab and IgG isotype control (100 μg/ml) and incubated with expanded and activated NK cells for additional 4hrs at 37°C at 20:1 effector: targets ratio. Cell killing was determined by DELFIA cell cytotoxicity assay kit. (N=3) **(C)** Raji, p=0.0002 and **(D)** Raji4RH, p=0.1. Data were shown in mean ±SD. (N=3).

Based on the differential phosphorylation of numerous proteins associated with NK cell-mediated cytotoxicity pathway (Figure 2 and Figure 3), we performed functional validation by directly comparing the cell killing of Raji and Raji4RH by activated and expanded NK cells with or without inhibition of MAPK1 protein using U0126 inhibitor after obinutuzumab compared to RTX treatment (Figure 6).

First, we verified the cytotoxicity pathway proteins differentially phosphorylated in Raji vs. Raji4RH after obinutuzumab compared to RTX treatment by western blot analysis. As shown in Figure 6A and 6B, RAF and ERK1 were differentially phosphorylated in Raji vs. Raji4RH treatment with obinutuzumab, although Raji4RH differentially phosphorylates RAF1 phosphorylation after RTX treatment compared to obinutuzumab (Figure 6A). Since expanded NK cells express high levels of CD16, an Fc receptor that mediates ADCC, we sought to determine if lytic NK activity against BL cell lines could be suppressed by MAPK1 inhibition in the presence of obinutuzumab or RTX (Figure 6C). We observed that MAPK1 inhibition in the presence of obinutuzumab reduced significantly the lytic effect of NK cells (p=0.00002) (Figure 6C). MAPK1 inhibition had no effect on the lytic activity of NK cells when Raji was treated with RTX. In contrast, MAPK1 inhibition had no effect on NK-cell lytic activity against Raji4RH treated with obinutuzumab or RTX (Figure 6D).

## DISCUSSION

We demonstrated that obinutuzumab compared to RTX differentially phosphorylates BCR, phagocytosis and cytotoxicity signaling pathways in RTX-sensitive and -resistant BL. Furthermore, we demonstrated that the knockdown of BCR signaling pathway protein, PLCG2, significantly enhanced BL proliferation and reduced cell death after obinutuzumab compared to RTX. Previously, we reported that obinutuzumab significantly enhanced apoptosis, ADCC activity and increased OS compared to RTX in BL and pre-B-ALL xenografted NSG mice [31]. We and others have previously observed that obinutuzumab compared to RTX differentially elicit several intracellular signaling BL pathways that result in reversing the chemo-resistant and immune-resistant phenotype to sensitive phenotypes [31, 37]. Thus, phenotypic responses of cancer cells to compounds that target intracellular kinase signaling are useful for the identification of novel markers of resistance or sensitivity to drugs that initiate the signaling network [38].

In this study, we have used an unbiased approach to analyze the phosphoproteomic profiles of RTX-sensitive (Raji) and RTX-resistant (Raji4RH) cell lines after obinutuzumab compared to RTX. The phoshoproteomic approach permitted identification and semi-quantification which facilitates detection of phosphorylated signaling pathways (Figure 2). The assembled data set of MOAC and pY-IP enrichments identified 978 unique phosphoproteins that were used to characterize signaling pathways differentially phosphorylated between sensitive and resistant RTX BL cell lines. Our study demonstrated differential phosphorylation of the BCR signaling, NK-cell-mediated cytotoxicity and Fc gammaR-mediated phagocytosis signaling pathways between obinutuzumab compared to RTX. Further, PLCG2 and ERK1 proteins highly phosphorylate after obinutuzumab compared to RTX treatment in Raji compared to Raji4RH, we selected these two proteins for functional validation.

The development, activation and programmed cell death of B cells require signals transduced through the BCR. Engagement of BCR by its cognate antigen leads to the formation of BCR micro clusters, to which many signaling molecules require to form “signalosome” [39]. One of the earliest events for the engagement of BCR is the phosphorylation of the immunoreceptor tyrosine-based activation motifs (ITAMs) by tyrosine kinases like, Lyn, LCK, which may further regulate signaling cascades of PLCG2, BTK and GSK3B [40].

Our study revealed phosphosignatures implicating BCR signaling in GC-derived BL (RTX-sensitive and -resistant) cell lines when treated with RTX or obinutuzumab. We identified 13 proteins in Raji and 10 in Raji4RH with phosphorylated tyrosine residues that contribute in the BCR signaling pathway following obinutuzumab compared to RTX treatment. These include four kinases (Lyn, LCK, BTK and GSK3B) and one enzyme PLCG2 (phospholipase C family catalyzes the hydrolysis of phospholipids) that could be a therapeutic target for RTX-resistant and -sensitive BL. Preclinical studies of BTK inhibitor, Ibrutinib, have shown promising results in different tumor cell lines [41-43]. Differential phosphorylation of PLCG2, BTK and Lyn was observed to be higher with obinutuzumab compared to RTX in Raji vs. Raji4RH as confirmed by both western blot as well as phosphoproteomic analysis.

BCR and pre-BCR signaling is mediated by a signalosome composed of Syk, BTK, B cell linker protein (BLINK or SLP65), and phospholipase C (PLC) G2. BTK and PLCG2 bind to BLINK by SH2 domain, which facilities activation of BTK and PLCG2 to mobilization of intracellular Ca+ ions [44]. Phosphorylated PLCG2 binds to activated RAS to promote the activation of ERK [40, 45].

These large differences in phosphorylation intensities of proteins among two cell lines are noteworthy and may be as important as loss of CD20 in RTX-resistant cell line Raji4RH, and may in part be secondary to low efficacy of obinutuzumab in Raji4RH cell lines as compared to Raji [24]. A recent study comparing 3D and 2D models of obinutuzumab compared to RTX in follicular lymphoma cell lines showed results that obinutuzumab displays enhanced efficacy compared to RTX in 3D model [46]. Although some of the EBV negative BL cell lines, BJAB and Ramos expressed baseline level of phospho-PLCG2, so this outcome may not apply to all BL [35].

We also recently demonstrated that glycoengineered type II CD20 antibody, obinutuzumab, was associated with a significant increase in ADCC compared to RTX against CD20 positive RTX-sensitive Raji and RTX-resistant (Raji2R and Raji4RH) and pre-B-ALL (U-698M) tumor targets [31]. In the present study we investigate the differential phosphorylation of proteins in NK-mediated cytotoxicity pathway RAF1/ERK1/MAPK1 and observed that approximately 8 proteins in Raji, while only 4 in Raji4RH, differentially phosphorylated after obinutuzumab compared to RTX. Silencing of MAPK1 pathway by a MAPK1 inhibitor (U0126) reduces cytotoxicity after obinutuzumab compared to RTX in Raji and suggests in part that NK-mediated cytotoxicity pathway is more sensitive to obinutuzumab compared to RTX [47]. Recently, it has been demonstrated that ADCC induced by RTX can be reduced by C1q binding to the Fc region that interferes with FcγR binding and may significantly decrease its impact on ADCC. Furthermore, type I CD20 antibodies were reported to show stronger CD20 internalization upon antibody binding compared to type II antibodies [15, 28]. These factors may in part contribute to enhance ADCC mediated by obinutuzumab compared to the type-I CD20 antibody, RTX [7, 29, 48].

In summary, our results demonstrate for the first time that obinutuzumab, a novel type-II anti-CD20 antibody obinutuzumab vs. RTX differentially phosphorylate specific BL protein signal transduction pathways that are involved in part regulating B-cell apoptosis and ADCC. These findings suggest that obinutuzumab should be considered as an alternative treatment in patients with relapsed/refractory aggressive BL, especially in those who fail RTX therapy [49]. More recently, Goede et al. demonstrated in patients with acute and untreated CLL, significantly increased PFS in patients receiving obinutuzumab and chlorambucil vs. RTX and chlorambucil [32]. Finally, these results offer insights into alternate therapeutic strategies to be considered in RTX and obinutuzumab treated BL.

## MATERIALS AND METHODS

### Cell lines

Human B-cell lymphoma Raji (CD20+) cell line was obtained from ATCC (CCL-86, CRL2629, Manassas, VA, USA); and RTX-resistant cell lines Raji2R and Raji4RH were generously supplied by Matthew J. Barth, MD, and Myron S. Czuczman, MD (Roswell Park Cancer Institute, Buffalo, NY, USA) [24]. Cell lines were maintained in RPMI 1640 media supplemented with 10% FCS and 2mM glutamine (Invitrogen, Grand Island, NY, USA) at 370C, 5% CO2. All cell lines were tested on a regular basis for cell morphology (by microscopic examination) and cell viability assay using alamar blue (Thermo Fisher Scientific, NJ, USA). The cell lines have not been authenticated since they were received in our laboratory.

### Antibodies and reagents

Obinutuzumab (GA101) was generously supplied by Hoffmann La Roche (Switzerland). RTX was purchased from Genentech Inc. (South San Francisco, CA, USA). Antibodies for flow cytometry, CD20-PE (Clone HI47), CD20-FITC (Clone HI47) and CD19-FITC (SJ25-C1), were obtained from Invitrogen (CA, USA). Western blot antibodies, phospho-PLC gamma2 (Tyr759, 150 KDa), total PLC gamma2 (150 KDa), phospho GSK3 Beta (Ser21/9, 46 KDa), total GSK3 Beta (46KDa), phospho BTK (Tyr223, 78KDa), total BTK (Clone D3H5, 77KDa), phospho Lyn (Tr507, 53, 56KDa) and total Lyn (C13F9,58KDa), phospho B- RAF (Ser445, 86KDa), total B- RAF (clone 55C6, 86KDa), phospho ERK1/2 (Thr202/Thyr204) (Clone D1.14.4E, 42, 44KDa), total ERK1/2 (42,44KDa), phospho LCK (Tyr505, 56KDa), total LCK (56KDa) and B-actin (clone D6A8, 45KDa) were obtained from Cell Signaling Technology (Danvers, MA, USA). CD20 (B9E9) antibody was obtained by Santa Cruz Biotechnology (Dallas, TX, USA). Annexin V: PE Apoptosis Detection Kit I was obtained from BD Biosciences (San Jose, CA, USA). Alamar blue cell viability reagent was purchased from Invitrogen (Grand Island, NY, USA). U0126 MEK1/2 inhibitor was purchased from Cell Signaling Technology (Danvers, MA, USA). PLC gamma2 and GSK3 Beta ON-TARGET plus siRNA were obtained from Dharmacon GE (Lafayette, CO, USA).

### Protein extraction and digestion

BL cells lines were incubated with obinutuzumab or RTX or IgG isotype control (100ug/ml, for 24hrs). Sixty million cells (Raji and Raji 4RH) were lysed in buffer containing 9 M urea/20 mM HEPES pH8.0/0.1% SDS and a cocktail of phosphatase inhibitors (sodium orthovanadate, sodium pyrophosphate and β-glycerophosphate). Protein samples were sonicated on ice then spun at 16,000 g for 10 min. For each sample, 6mg of protein were reduced with 4.5 mM DTT for 30 min at 60°C and then alkylated with 10 mM iodoacetamide for 30 min at room temperature in the dark. Samples were diluted 5-fold with 20 mM HEPES and then digested with trypsin overnight at 37°C using an enzyme-to-protein ratio of 1/50 (w/w). After acidification with 1% trifluoroacetic acid, samples were desalted on a C18 cartridge (Sep-Pak plus C18 cartridge, Waters), then purified peptides were dried before further processing.

### Phosphopeptide enrichment

MOAC was performed to enrich phosphorylated peptides and reduce the sample complexity prior to tyrosine-phosphorylated peptide immunoprecipitation. We used titanium dioxide (TiO_2_) microparticles from GL Sciences Inc. (Titansphere® Phos-TiO, GL Sciences Inc.) applying a TiO_2_ microparticles-to-protein ratio of 6/1 (w/w). Briefly TiO_2_ microparticles were conditioned with 100 μl buffer A (80% ACN/0.4% TFA), then equilibrated with 100 μl buffer B (75% buffer A/25% lactic acid). Peptides were solubilized with 200 μl buffer A and mixed with 400 μl buffer B then loaded twice on TiO_2_ microparticles. Microparticles were washed 2 times with buffer B and 3 times with buffer A. Hydrophilic phosphopeptides were eluted with 5% ammonium hydroxide solution and hydrophobic phosphopeptides were eluted with 5% pyrrolidine solution. After elution, peptides were dried using a SpeedVac. The equivalent of 5 mg of protein was further enriched for phosphorylated tyrosine peptides by overnight immunoprecipitation using a cocktail of anti-phosphotyrosine antibodies (4G10, Millipore/PT-66, Sigma/p-Tyr-100, Cell Signaling Technology). After elution, phosphotyrosine peptides were dried.

### Mass spectrometry analysis

Ammonium hydroxide and pyrrolidine eluents were dried (SpeedVac) and reconstituted in 25 μl sample loading buffer (0.1% TFA/2% acetonitrile). Eluents from phosphotyrosine immunoprecipitation were dried and reconstituted in 35 μl of the loading buffer. An LTQ Orbitrap XL (ThermoFisher) in-line with a Paradigm MS2 HPLC (Michrom bioresources) was employed for acquiring high-resolution MS and MS/MS data. Ten microliters of the phospho-enriched peptides were loaded onto a sample trap (Captrap, Bruker-Michrom) in-line with a nano-capillary column (Picofrit, 75 μm i.d.x 15 μm tip, New Objective) packed in-house with 10 cm of MAGIC AQ C18 reverse phase material (Michrom Bioresource). Two different gradient programs, one each for MOAC and phosphotyrosine immunoprecipitation samples, were used for peptide elution. For MOAC samples, a gradient of 5-40% buffer B (95% acetonitrile/1% acetic acid) in 135 min and 5 min wash with 100% buffer B followed by 30 min of re-equilibration with buffer A (2% acetonitrile/1% acetic acid) was used. For phosphotyrosine immunoprecipitation samples, which were a much less complex mixture of peptides, 5-40% gradient with buffer B was achieved in 75 min followed by 5 min wash with buffer B and 30 min re-equilibration. Flow rate was ∼0.3 μl/min. Peptides were directly introduced into the MS using a nano-spray source. Orbitrap was set to collect 1 MS scan between 400-2000 m/z (resolution of 30,000 @ 400 m/z) in orbitrap followed by data dependent CID spectra on top 9 ions in LTQ (normalized collision energy ∼35%). Dynamic exclusion was set to 2 MS/MS acquisitions followed by exclusion of the same precursor ion for 2 min. Maximum ion injection times were set to 300 ms for MS and 100 ms for MS/MS. Automatic Gain Control (AGC) was set to 1xe6 for MS and 5000 for MS/MS. Charge state screening was enabled to discard +1 and unassigned charge states. Technical duplicate data for each of the MOAC elutions (ammonium hydroxide and pyrrolidine) and triplicate data for the phosphotyrosine immunoprecipitation samples were acquired.

### Bioinformatics analysis

RAW files were converted to mzXML using msconvert. The MS data was then searched with the Swissprot Human taxonomic protein database (2013Jan09 release) appended with common proteomics contaminants and reverse sequences as decoys. Searches were performed with X!Tandem (version 2010.10.01.1) using the k-score plugin. For all searches the following search parameters were used: A parent monoisotopic mass error window of 50 ppm was used. The fragment ion error window was 0.8 Da. Searches were performed allowing for up to 2 missed tryptic cleavages. We allowed for the potential modifications of oxidation of Methionine (+15.9949@M), carbamidomethylation of Cysteine (+57.0214@C), and phosphorylation of Serine, Threonine, and Tyrosine (+79.9663@[STY]). The search results were then post-processed through the Trans-Proteomic Pipeline (TPP) using PeptideProphet and ProteinProphet [24-26].

Spectral counts for the TPP results were obtained for each cell line using the spectral counting software ABACUS. Tyrosine-enrichment data were processed through ABACUS separately from the serine and threonine data. ABACUS was run in verbose mode to report all protein identifiers. Protein groups were filtered to only retain those proteins with a ProteinProphet probability greater than 0.7. Only peptides with a probability greater than 0.8 and containing a phosphorylation on a serine, threonine or tyrosine were considered. Label-free spectral counting was used from the ABACUS output in all further analysis to quantify the relative abundance of phosphorylated peptides/proteins. All ABACUS results were then loaded into a relational database for further analysis. The proteins reported by ABACUS were then manually curated to select the best representative protein identifier from among ambiguous cases.

### Cell proliferation assay

BL cell lines were treated with 100 μg/ml obinutuzumab, RTX or isotype control as described above. Alamarblue dye was added at 10% sample volume, and incubated 1-4 hrs at 37°C. BL cell viability was determined by fluorescence spectrophotometer at 580nm excitation emission at 600 nm. Cell proliferation was calculated as percentage of fluorescence of cells with respect to untreated control cells, after subtracting for background fluorescence in absence of cells.

### Small interfering siRNA

Raji and Raji 4RH cells were transfected with on target plus genome pools SiRNA (Dharmacon GE, Lafayette, CO, USA) for PLC gamma 2 or siCONTROL at 2 nM using lipofectamine 2000 (Thermo Fisher Scientific, Walthem, MA). Knockdown of PLC gamma 2 and GSK3 Beta protein levels were confirmed by western blotting after 24-48 h of incubation.

### IgM stimulation

In some cases, cell lines were stimulated for 48 hrs prior with treatment of drugs (RTX or obinutuzumab or silencing with PLCG2), with or without anti-IgM antibody (50 ug/ml, Invitrogen, Grand Island, NY). Stimulated cells were used for cell proliferation or apoptosis assay as described above.

### SDS-PAGE and Western blot

BL cells were lysed in RIPA buffer containing protease inhibitor cocktail (Roche). Total protein (40μg) were suspended in Laemmli sample buffer, boiled, and subjected to SDS polyacrylamide gel electrophoresis on 10% gels. Proteins were transferred to polyvinylidine difluoride membranes (PVDF) by electroblotting and the membranes were incubated in primary antibody with 2% blocking agent overnight. Blot was stripped and re-probes with beta-actin antibody.

### Cytotoxicity assay

NK cells were isolated and expanded as previously described [31] from buffy-coat products from normal healthy adult donors after informed consent from the New York Blood Center and mononuclear cells (PBMC) were isolated by Ficoll-Paque (Amersham Biosciences, Piscataway, NJ, USA) density gradient separation. Expanded NK cells have more cytotoxic activity compared to IL-2 generated NK as we have previously described [31]. After 6 days of expansion NK cells were harvested, washed, counted and re-cultured in 10%-RPMI medium with IL-2 for additional 2-3 days. Cytotoxicity was determined using DELFIA EuTDA cytotoxicity assay as we have previously described [31].

### Statistical analyses

Statistical significance between treatment groups and controls were determined by Student's t-test mean ± SD. Experiments were performed in triplicate for 3 independent experiments. A p-value of <0.05 was considered as having statistical significance for all *in vitro* studies.

## SUPPLEMENTARY MATERIALS FIGURES



## Abbreviations

7-AAD: 7-Aminoactinomycin D
ADCC: antibody-dependent cell cytotoxicity
AGC: Automatic Gain Control
BCR: B cell antigen receptor
B-NHL: B-cell NHL
BL: Burkitt lymphoma
BTK: Bruton tyrosine kinase
CLL: chronic lymphocytic leukemia
DAVID: Database for Annotation, Visualization and Integrated Discovery
DLBCL: diffuse large B-cell lymphoma
EBV: Epstein Barr virus
ERK: extracellular signal-regulated kinase
Fc: fragment crystallizable
IgG: immunoglobulin G
mAbs: monoclonal antibodies
ITAM: immunoreceptor tyrosine-based activation motifs
MOAC: metal oxide affinity chromatography
MS: mass-spectrometry
NHL: Non-Hodgkin lymphoma
NK: natural killer
NSG: non-obese diabetic severe combined immunodeficiency gamma
OS: Overall survival
PFS: progression free survival
PLCG2KD: *PLCG2* knockdown
pre-B-ALL: precursor B-cell acute lymphoblastic leukemia
RTX: Rituximab
SCID: severe combined immunodeficiency
TiO_2_: titanium dioxide
TPP: Trans-Proteomic Pipeline
