# Supplementary material for: A comparative global phosphoproteomics analysis of obinutuzumab (GA101) versus rituximab (RTX) against RTX sensitive and resistant Burkitt lymphoma (BL) demonstrates differential phosphorylation of signaling pathway proteins after treatment
Source: Oncotarget. 2017 Dec 9;8(69):113895–909. doi: 10.18632/oncotarget.23040 (PMC5768372; doi:10.18632/oncotarget.23040)
Supplement: Supplementary file 1 [file oncotarget-08-113895-s001.pdf]

A comparative global phosphoproteomics analysis of obinutuzumab (GA101) versus rituximab (RTX) against RTX sensitive and resistant Burkitt lymphoma (BL) demonstrates differential phosphorylation of signaling pathway proteins after treatment

SUPPLEMENTARY MATERIALS

Cell death assay

Raji and Raji4RH cell lines were seeded in 96 well plates at 10^6/ml, with obinutuzumab (100 μg/ml), RTX (100μg/ml) or human polyclonal IgG (isotype control) with or without silencing of PLC gamma 2 and incubated at 37°C with 5% CO2 for 24 hours. Cells were washed, re-suspended in 10% RPMI and stained with annexin V-PE /7-Aminoactinomycin D (7-AAD) to detect loss of plasma membrane integrity and with CD19-FITC. Cell death was determined by flow cytometry (MACS-quant, Miltenyi Biotec, CA). Cells were evaluated within the lymphocyte gate; approximately 20,000 events were captured and analyzed for each sample.

Immunohistochemistry: Raji and Raji 4RH cells were seeded in Lab tak chamber slide (Nalge Nunc, Roskilde, Denmark) with obinutuzumab or RTX 100μg/ml for 24 hrs. Cells were washed with PBS and blocked with serum for 1 hrs at room temperature. Incubate with primary antibody (P-PLCG2) 1hr at room temperature and rinsed with PBS-Tween-20. Cells were further incubated with secondary antibody conjugated with alexa-488 (Life technology, Waltham, MA. USA) for 1 hr and mount with mounting medium (Vector Laboratory, Burlingame, CA. USA).

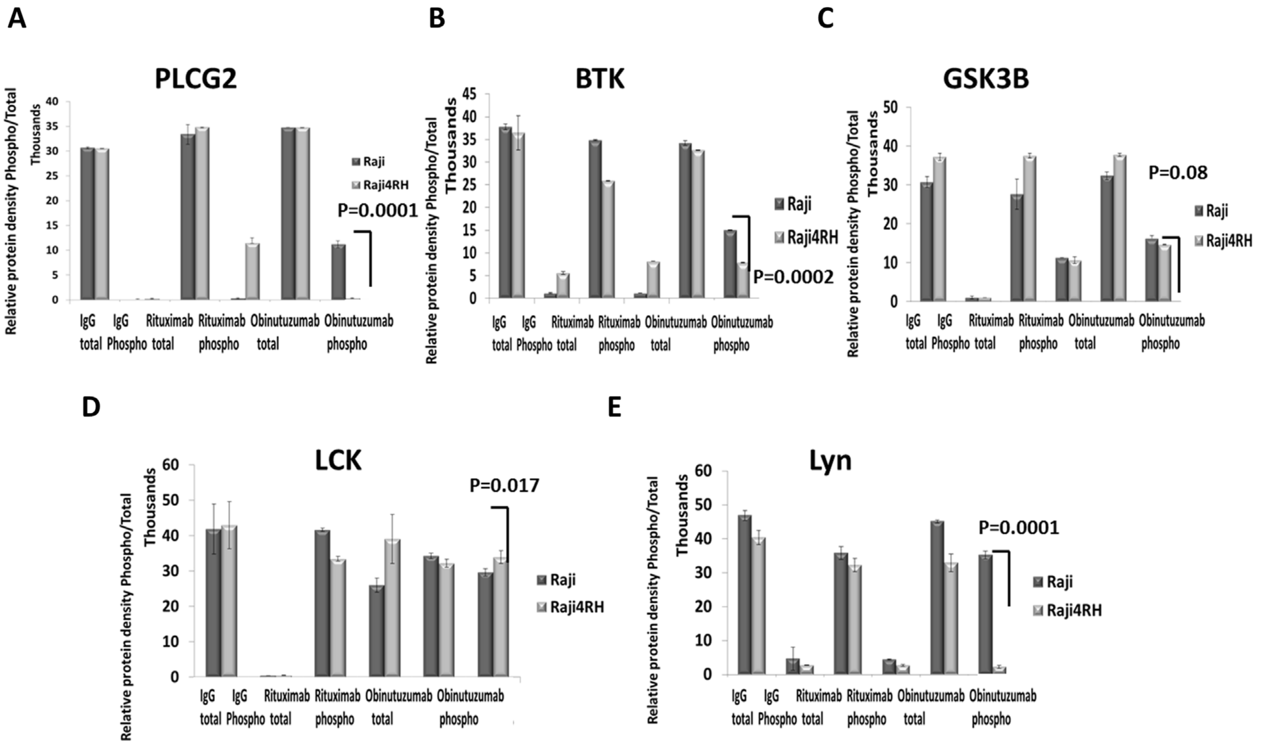

Supplementary Figure 1: Phosphorylation of proteins involved in BCR signaling pathway are differentially altered after obinutuzumab (Obit) vs. rituximab (RTX). Phosphorylation of proteins involved in BCR signaling pathways were **(A)** PLCG2, (p=0.001), **(B)** BTK (p=0.002), **(C)** GSK3B (p=NS), **(D)** LCK,(p=0.017) and **(E)** Lyn, (p=0.00001) in Raji and Raji4RH BL cell lines. Obinutuzumab vs. rituximab treated cells were lysed and subjected to SDS polyacrylamide gel electrophoresis. Relative protein density was quantified by Image J software. Data are represented as the mean ± SD (N=3).

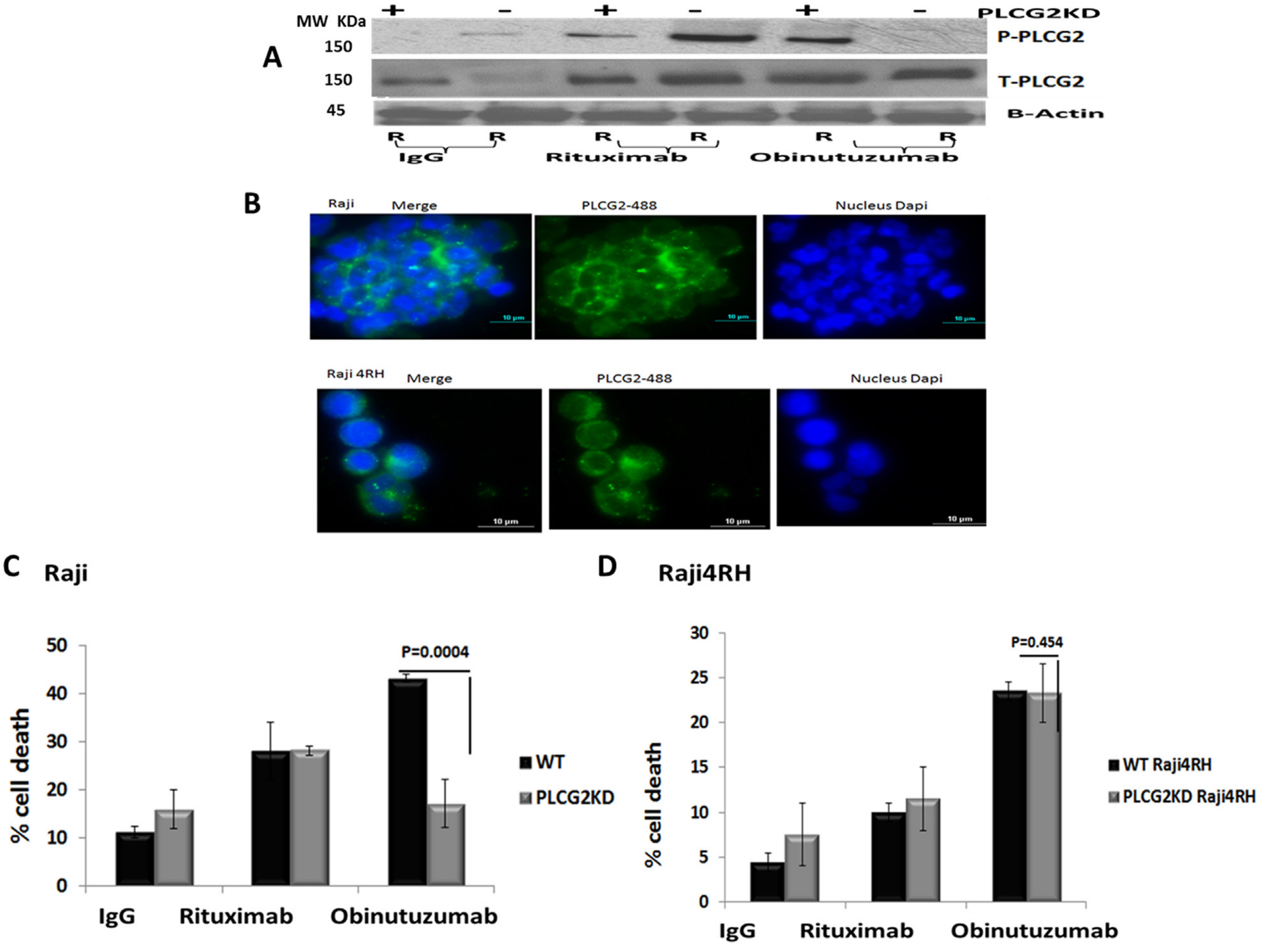

Supplementary Figure 2: PLCG2 Expression after obinutuzumab (Obit) vs. rituximab (RTX) in Raji vs. Raji4RH: **(A)** PLCG2 expression in Raji vs. Raji4RH by western blot. **(B)** obinutuzumab treated cells were stained with p-PLCG2-488 Alexa antibody and image by Nikon Eclipse 90i (Melville, NY. USA). **(C)** Raji and **(D)** Raji4RH cells were treated with MAPK1 inhibitor or untreated and further incubated with obinutuzumab or rituximab for direct cell death. Percentage of cell death was analyzed by Annexin V /7AAD staining (P=0.0004 and P=0.45, respectively). Data were shown in mean ±SD. (N=3).
